# The association between dietary consumption habits and psoriasis: a two-sample Mendelian randomization study

**DOI:** 10.3389/fnut.2024.1405663

**Published:** 2024-10-23

**Authors:** Lu Minghui, Gao Changyong, Zhang Runtian, Li Jianhong, Yuan Lingling, Chen Xi

**Affiliations:** ^1^Dongzhimen Hospital, Beijing University of Chinese Medicine, Beijing, China; ^2^Tongzhou District Branch, Dongzhimen Hospital, Beijing University of Chinese Medicine, Beijing, China

**Keywords:** psoriasis, dietary consumption habits, Mendelian randomization, dry fruit, GWAS

## Abstract

**Background:**

Psoriasis is a chronic inflammatory skin disease characterized primarily by erythema and scales, having a wide-ranging impact globally. Previous studies have suggested that dietary consumption habits may influence psoriasis. The objective of this study was to determine the causal relationship between dietary consumption habits and psoriasis using the Mendelian Randomization (MR) approach.

**Methods:**

SNP data for 29 dietary consumption habits and psoriasis were obtained from the GWAS catalog database and the FinnGen database, respectively. The Mendelian Randomization analysis was performed using R software, with the 29 dietary consumption habits as the exposure factors and psoriasis as the outcome. Three MR analysis methods—Inverse Variance Weighted (IVW), Weighted Median Estimator (WME), and MR-Egger regression—were employed to study the causal relationship between dietary consumption habits and psoriasis.

**Results:**

The IVW analysis indicated an OR (95%CI) of 0.065 (0.008–0.555), *p* = 0.012, demonstrating a negative correlation between the consumption of dried fruit and psoriasis.

**Conclusion:**

Among the 29 dietary consumption habits analyzed, the intake of dried fruits is a protective factor against psoriasis. Therefore, it is clinically advisable to appropriately increase the intake of dried fruits among patients with psoriasis, serving as a nutritional therapy method in conjunction with pharmacological treatment.

## 1 Introduction

Psoriasis is a chronic inflammatory skin disease characterized mainly by erythema and scales, representing an immune-mediated condition against a genetic background. The global prevalence among adults ranges from 0.09 to 11.43%, and between 0.0 to 1.3% in children, with an average prevalence rate of 2% (1), affecting the mental and physical health of approximately 125 million people worldwide (2). The World Health Organization has recognized this disease as a global issue, significantly disrupting the normal social interactions of patients, increasing their economic burden, and reducing their quality of life.

As psoriasis gradually becomes a burdensome global medical condition (3, 4), identifying potential risk factors triggering the disease has increasingly become a focus of attention. Infections, dysbiosis of the gut microbiome, psychological stress, and drugs are common risk factors for psoriasis, usually interacting with other risk factors, including genetic predispositions (5). In recent years, there has been a growing interest in exploring the association between dietary factors and psoriasis to determine whether they act as risk or protective factors. To date, more than one study has investigated the relationship between dietary consumption habits and psoriasis. Patients with obesity and psoriasis who followed a low-calorie diet of ≤1,000 kcal/day for 8 weeks exhibited a significantly greater reduction in the average Psoriasis Area and Severity Index (PASI) score compared to the control group (84% vs. 69%) (6). Studies by others (7) have similarly found that, under the same Mediterranean diet, the intake of berries, olive oil, nuts, and eggs by psoriasis patients was significantly lower than that of the control group, with psoriasis patients consuming more dairy products and soft drinks. However, due to the susceptibility of clinical case–control studies to confounding factors, the association between dietary consumption habits and psoriasis remains not very clear.

Mendelian randomization is a research method that examines the relationship between exposure factors and outcomes through genetic variants associated with the exposure (8). Compared to randomized controlled trials and case–control studies, it offers broad applicability, reduced confounding bias, ethical compliance, and low cost (9). Previous studies have indicated that low-calorie, Mediterranean, and restricted protein/vegetarian diets may all be beneficial dietary patterns for improving psoriasis (10). This study employs the two-sample Mendelian randomization approach to test the causal relationship between 29 dietary consumption habits and psoriasis, providing a reference for developing scientific dietary strategies for the prevention and improvement of psoriasis.

## 2 Materials and methods

### 2.1 Acquisition of Instrumental Variables

The keywords “food consumption” and “psoriasis” were used to search in the GWAS catalog database^1^ and FinnGen database.^2^ The SNP dataset for dietary consumption habits was derived from the study by Pirastu N et al. in the GWAS catalog database. The dataset is presented in Table 1 (11); the SNP dataset for psoriasis was obtained from the FinnGen database, including 407,876 samples, of which 10,312 were cases and 397,564 controls (12). All sample populations were of European ancestry to avoid potential biases due to racial factors. All data used have been published in the public domain and have obtained informed consent from participants. The research protocol was approved by local, regional, or institutional ethics committees, and no additional ethical approval was required.

**Table 1:** Descriptive information of 29 lifestyle and dietary factors.

Food consumption	GWAS ID	Sample size (overall or case/control)	Ancestry	Consortium	Year
Beef consumption	GCST90096901	241,092	European	N/A	2022
Beer or cider consumption	GCST90096902	168,237	European	N/A	2022
Spread on bread consumption	GCST90096903	445,506	European	N/A	2022
Bread consumption	GCST90096904	438,853	European	N/A	2022
Champagne or white wine consumption	GCST90096905	175,549	European	N/A	2022
Cheese consumption	GCST90096906	365,842	European	N/A	2022
Cooked vegetables consumption	GCST90096907	435,417	European	N/A	2022
Decaffeinated coffee consumption	GCST90096908	62,072	European	N/A	2022
Dried fruit consumption	GCST90096909	409,125	European	N/A	2022
Drink temperature	GCST90096910	444,097	European	N/A	2022
Fortified wine consumption	GCST90096911	31,836	European	N/A	2022
Fresh fruit consumption	GCST90096912	433,186	European	N/A	2022
Ground coffee consumption	GCST90096913	72,276	European	N/A	2022
Instant coffee consumption	GCST90096914	180,764	European	N/A	2022
Lamb consumption	GCST90096915	189,984	European	N/A	2022
Percentage fat in milk consumption	GCST90096916	411,503	European	N/A	2022
Non-oily fish consumption	GCST90096917	318,136	European	N/A	2022
Oily fish consumption	GCST90096918	297,881	European	N/A	2022
Pork consumption	GCST90096919	187,202	European	N/A	2022
Poultry consumption	GCST90096920	399,716	European	N/A	2022
Processed meat consumption	GCST90096921	312,220	European	N/A	2022
Red wine consumption	GCST90096922	211,628	European	N/A	2022
Salad/raw vegetables consumption	GCST90096923	422,542	European	N/A	2022
Added salt consumption	GCST90096924	323,995	European	N/A	2022
Spirits consumption	GCST90096925	118,477	European	N/A	2022
Tea consumption	GCST90096926	434,171	European	N/A	2022
Vegetarianism	GCST90096927	442,589	European	N/A	2022
Water consumption corrected for coffee	GCST90096928	400,642	European	N/A	2022
Water consumption	GCST90096929	445,799	European	N/A	2022

GWAS, Genome-Wide Association Studies; N/A, not applicable.

### 2.2 Selection of instrumental variables

**Figure 1:**
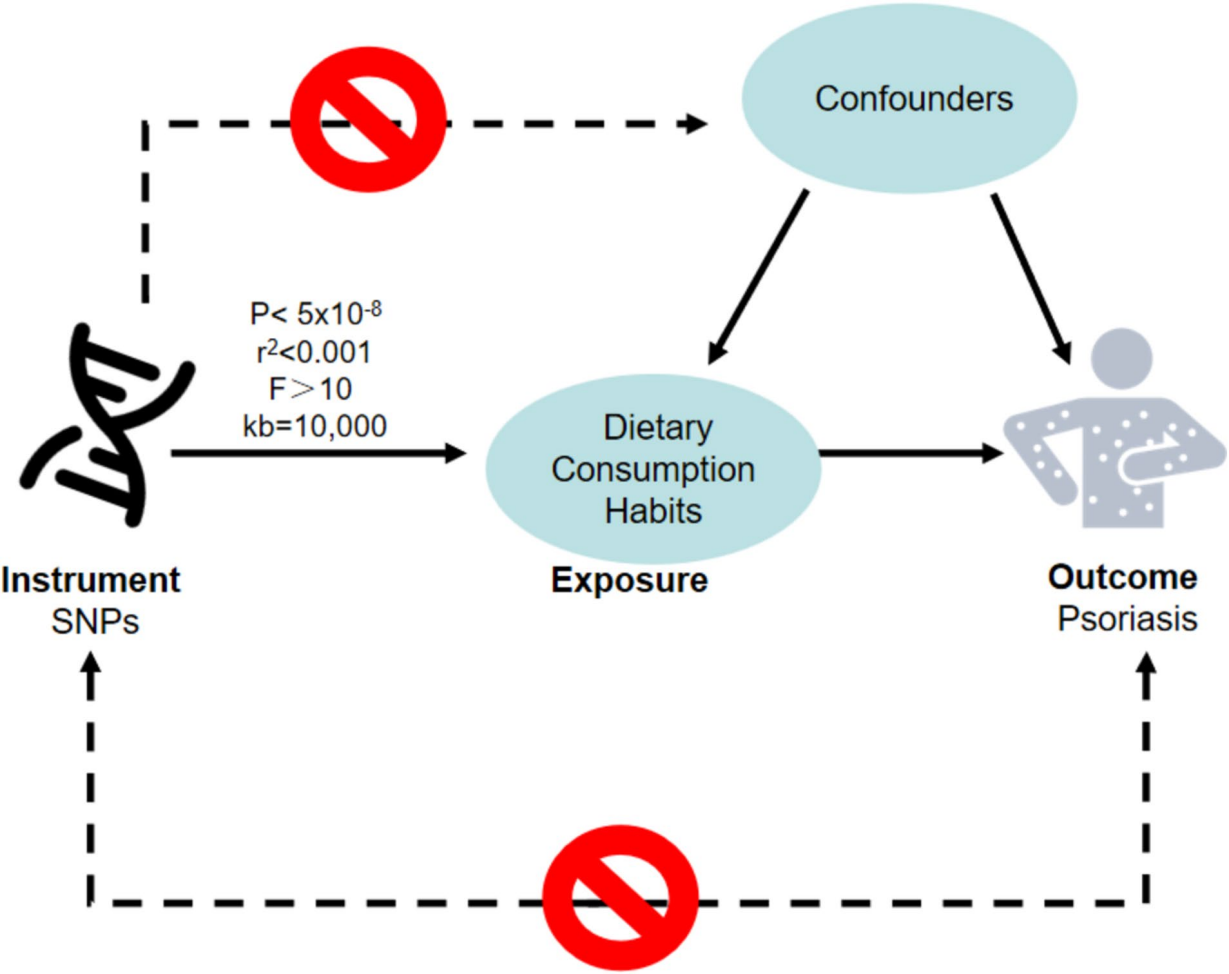
The hypotheses that must be met for Mendelian randomization studies. A node represents a variable, a solid line represents MR analysis processes and can only influence the outcome by exposure and a dashed line represents a causal relationship is prohibited. MR, Mendelian randomization; SNPs, single nucleotide polymorphisms.

Since Mendelian Randomization (MR) requires that the chosen instrumental variables meet three criteria: the instrumental variables must be strongly associated with the exposure factor, independent of confounding factors, and affect the outcome variable only through the exposure factor. We first set *p* < 5 × 10^−8^ and *F* > 10 to select SNPs strongly associated with the exposure factor, thereby avoiding the impact of weak instrumental variables. Secondly, we set an r^2^ threshold of 0.001 and a base pair region range of 10,000 kb to ensure there is no linkage disequilibrium in the results. Finally, the PhenoScanner database was utilized to exclude confounding factors (Figure 1).

### 2.3 Mendelian randomization analysis

**Figure 2:**
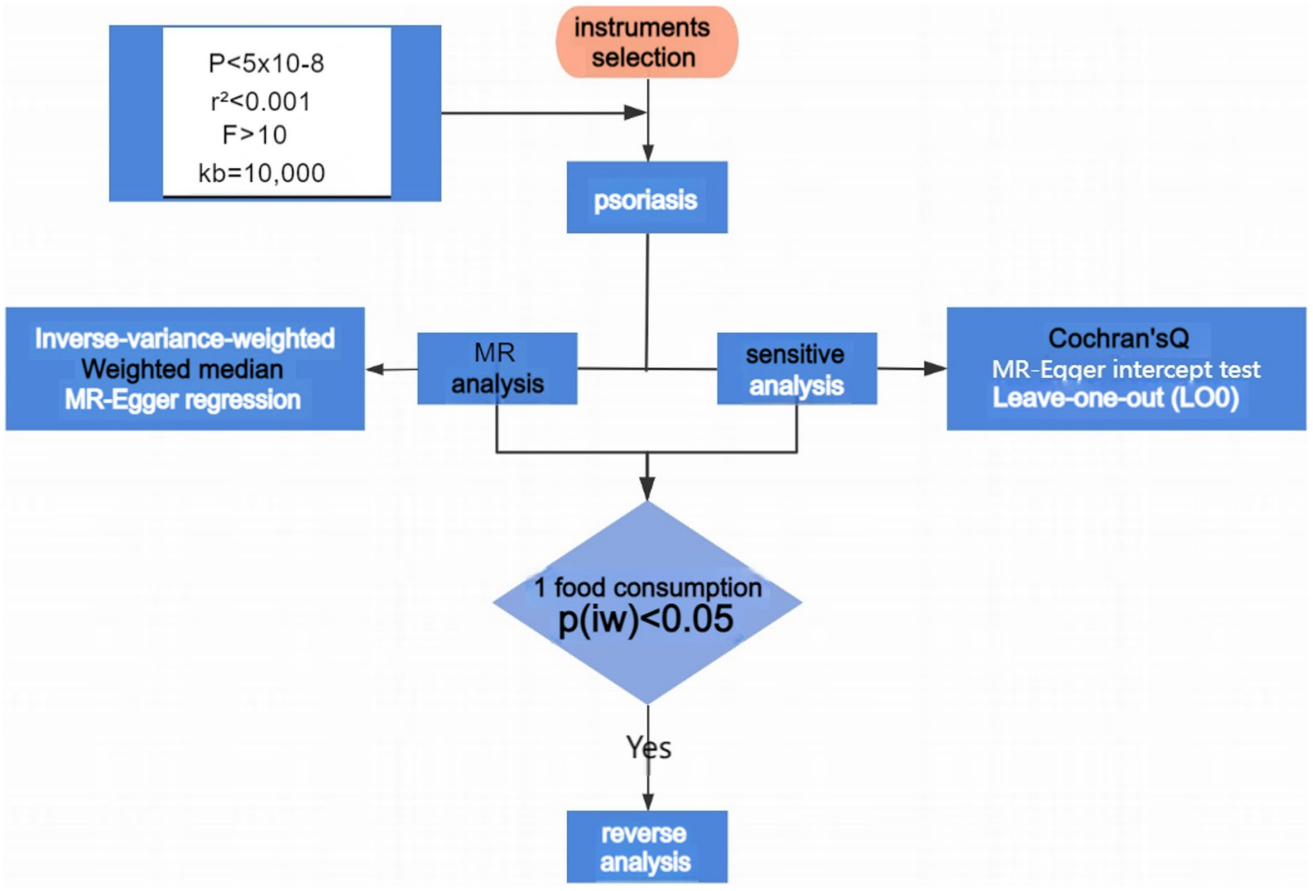
Flowchart of MR analyses in this study. MR, Mendelian randomization; IVW, inverse variance weighted.

This study employed the “TwoSampleMR” package in R 4.2.1, integrating three Mendelian Randomization analysis methods to analyze the association between 29 dietary consumption habits and psoriasis. These methods are the Inverse-Variance Weighted (IVW) method, MR-Egger regression, and the Weighted Median Estimator (WME), with the IVW results generally serving as the primary reference, while the other two methods provide secondary evidence. Heterogeneity was tested using the Inverse-Variance Weighted and MR-Egger methods combined with Cochran’s Q test. A *p*-value <0.05 indicates the presence of heterogeneity, which can impact the analysis results to some extent. The intercept obtained through the MR-Egger method was further analyzed to test for horizontal pleiotropy. If *p* < 0.05, the data exhibit pleiotropy, and the analysis results cannot meet the basic requirements of Mendelian Randomization. Finally, a leave-one-out approach was employed to sequentially exclude each SNP and analyze the remaining SNPs to identify any with a significant impact on the results. Procedures and methods as seen in Figure 2.

## 3 Results

### 3.1 The impact of dietary consumption habits on psoriasis MR analysis

After relevant screening, 12 dietary habits with fewer than 3 SNPs were removed from the dietary consumption habits sample dataset, leaving 17 dietary habits with a larger number of SNPs (Table 2).

**Table 2:** The selected diet habits (SNPs number greater than or equal to 3).

Exposure	Outcome	nSNP	IVW-derived *p* value	OR (95%CI)	MR-Egger- derived P value	OR (95%CI)	WM-derived P value	OR (95%CI)
GCST90096893	Psoriasis	79	0.415	1.092 (0.884–1.349)	0.671	1.191 (0.533–2.658)	0.138	1.232 (0.935–1.624)
GCST90096894	Psoriasis	30	0.374	1.098 (0.893–1.350)	0.591	1.118 (0.747–1.673)	0.414	1.091 (0.885–1.346)
GCST90096895	Psoriasis	95	0.701	1.049 (0.823–1.337)	0.579	0.749 (0.270–2.074)	0.607	0.918 (0.663–1.272)
GCST90096902	Psoriasis	119	0.011	2.096 (1.183–3.714)	0.207	0.187 (0.014–2.497)	0.083	1.919 (0.919–4.005)
GCST90096903	Psoriasis	55	0.393	0.559 (0.147–2.122)	0.162	10.837 (0.402–292.102)	0.585	0.599 (0.095–3.777)
GCST90096904	Psoriasis	118	0.438	0.907 (0.710–1.160)	0.946	1.032 (0.424–2.507)	0.640	0.927 (0.677–1.271)
GCST90096905	Psoriasis	44	0.587	1.382 (0.430–4.441)	0.963	0.914 (0.020–41.213)	0.279	2.096 (0.548–8.011)
GCST90096906	Psoriasis	115	0.032	0.245 (0.067–0.887)	0.959	0.875 (0.005–149.703)	0.092	0.232 (0.042–1.271)
GCST90096907	Psoriasis	84	0.878	1.093 (0.353–3.383)	0.469	0.205 (0.003–14.629)	0.946	1.051 (0.249–4.429)
GCST90096908	Psoriasis	19	0.928	1.057 (0.320–3.486)	0.631	0.402(0.010–15.538)	0.518	0.654 (0.181–2.364)
GCST90096909	Psoriasis	169	0.000	0.477 (0.322–0.705)	0.399	0.490(0.094–2.556)	0.382	0.801 (0.487–1.317)
GCST90096916	Psoriasis	69	0.012	0.065 (0.008–0.555)	0.487	0.084(0.000–87.150)	0.084	0.098 (0.007–1.370)
GCST90096917	Psoriasis	31	0.892	0.924 (0.294–2.899)	0.158	16.623(0.373–740.958)	0.793	0.826 (0.198–3.450)
GCST90096921	Psoriasis	83	0.991	1.008 (0.276–3.685)	0.336	0.041 (0.000–26.690)	0.644	0.661 (0.114–3.831)
GCST90096922	Psoriasis	40	0.944	1.047 (0.293–3.742)	0.541	0.232 (0.002–24.142)	0.937	1.065 (0.225–5.036)
GCST90096928	Psoriasis	194	0.432	0.955 (0.852–1.071)	0.360	0.791 (0.479–1.306)	0.958	1.004 (0.869–1.160)
GCST90096929	Psoriasis	229	0.413	0.957 (0.861–1.064)	0.615	1.094 (0.771–1.552)	0.824	0.983 (0.845–1.144)

SNPs, single nucleotide polymorphisms; WM, weighted median; OR, odds ratio; CI, confidence interval.

**Figure 3:**
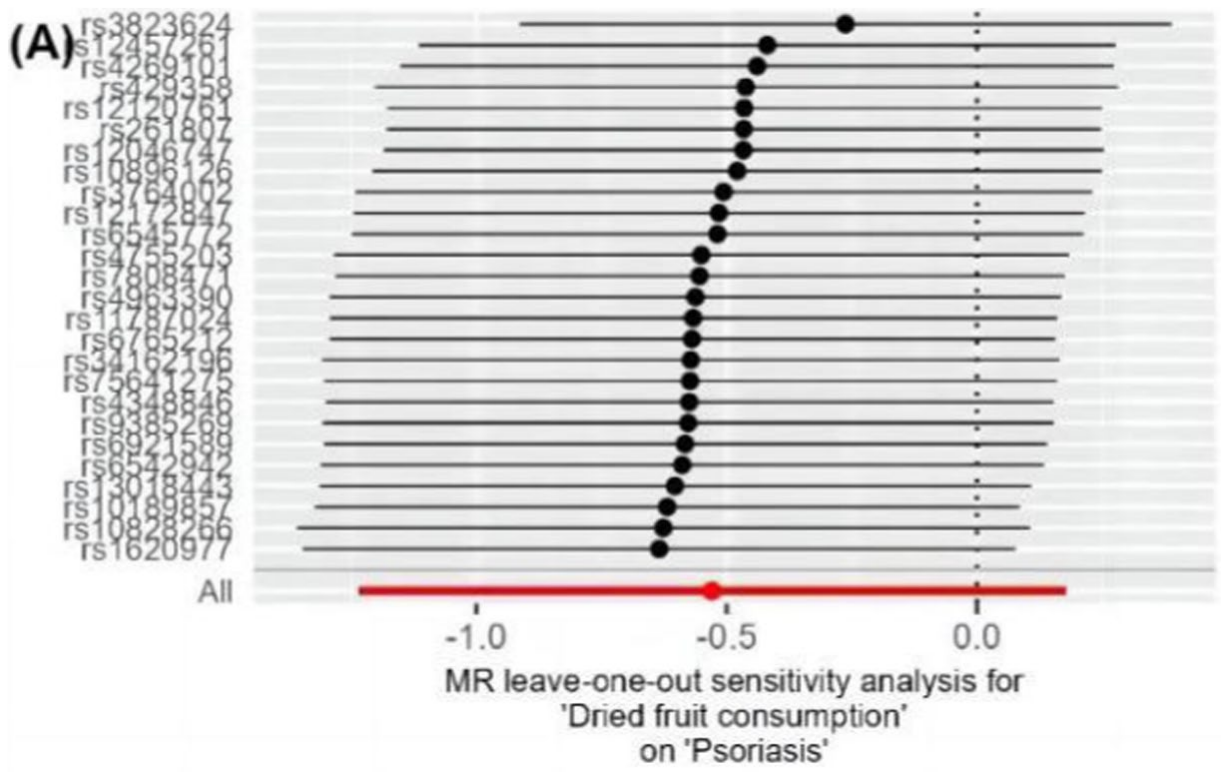
Leave-one-out analysis results of dietary habits on psoriasis. Forest plots of the “leave-one-out” sensitivity analyses to demonstrate the impact of individual SNPs on the results. The x-axis shows MR “leave-one-out” sensitivity analyses for dried fruit intake on psoriasis. The y-axis shows the analyses for the effect of “leave-one-out” of SNPs on psoriasis. The black point on the bottom line of each panel indicates the IVW estimate using all SNPs. MR, Mendelian randomization; SNP, single nucleotide polymorphism; IVW, inverse variance weighted.

**Figure 4:**
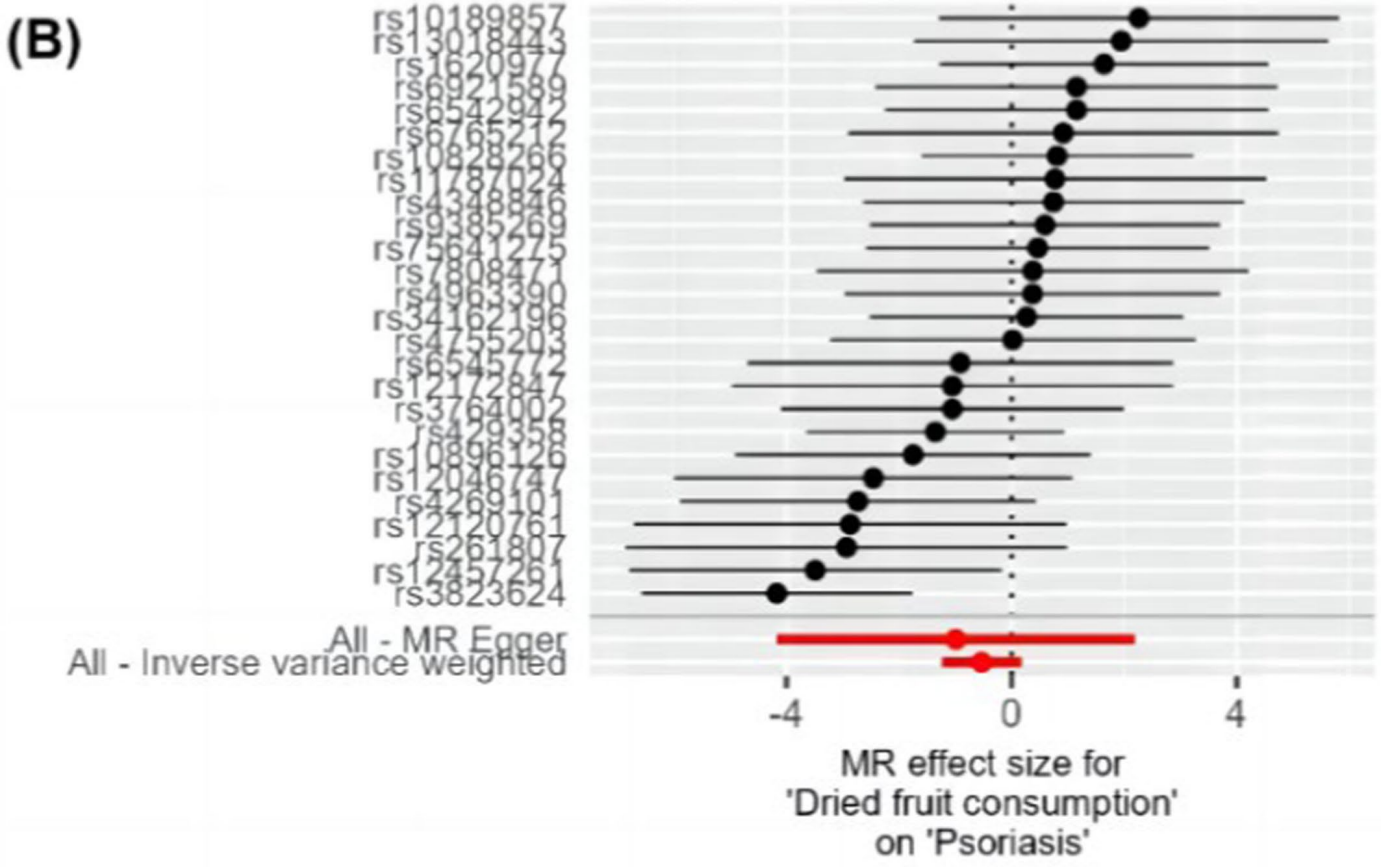
Forest plot of MR analysis of dietary habits on psoriasis. The vertical axis represents SNPs, and the horizontal axis represents effect values. Black dots and black horizontal lines represent the effect values and their fluctuation ranges for each SNP. Red dots and red horizontal lines represent the combined effect values and their fluctuation ranges obtained through MR Egger and IVW methods. Values greater than 0 indicate that the exposure factor is a risk factor, and values less than 0 indicate that the exposure factor is a protective factor. MR, Mendelian randomization; SNP, single nucleotide polymorphism; IVW, inverse variance weighted.

**Figure 5:**
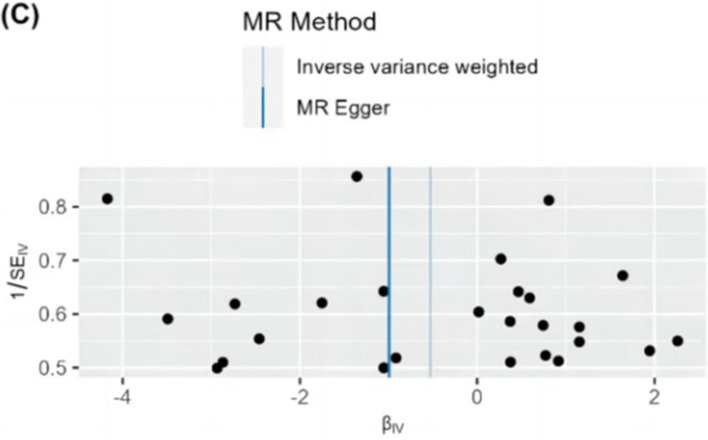
Funnel plot of MR analysis of dietary habits on psoriasis. Black dots represent SNPs. The MR estimate for each SNP is plotted against its minor-allele frequency corrected association with dried fruit intake. The vertical lines show the results of IVW or MR-ER using all SNPs. If the black dots on both sides of the vertical line are roughly symmetrical, it indicates that the results are homogenous. IVW, inverse variance weighted; MR, Mendelian randomization; SNP, single nucleotide polymorphism.

**Figure 6:**
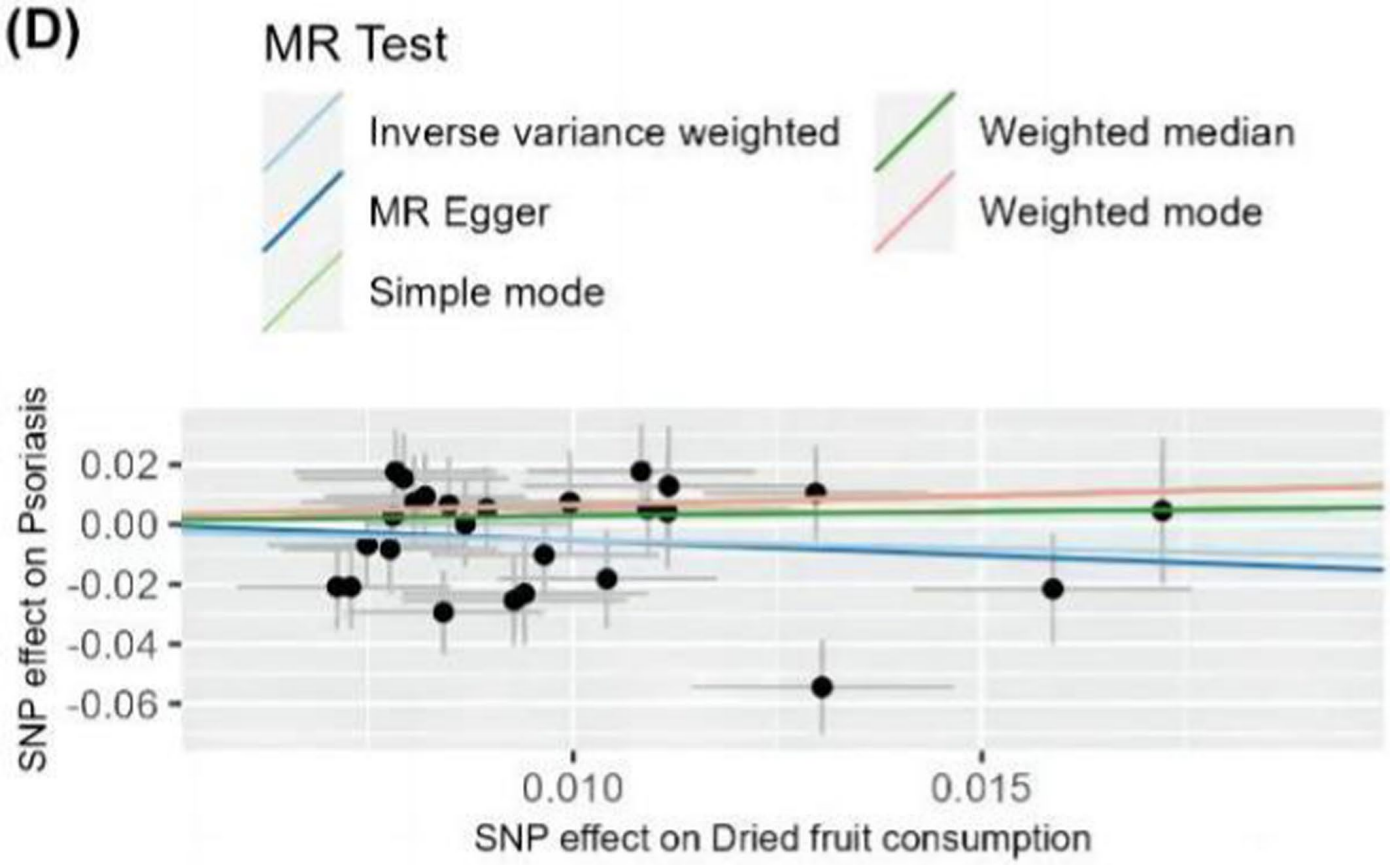
Scatter plot of MR analysis of dietary habits on psoriasis. Scatter plots were used to visualize the causal effect between dried fruit intake and psoriasis using five MR methods. Black dots represent SNPs, and the cross lines indicate the fluctuation range of the effect values for each SNP. The x-axis shows the SNP effect and SE on dietary factors. The y-axis shows the SNP effect and SE on psoriasis. The regression lines for the inverse-variance weighted (IVW) method, the MR-Egger regression method, the weighted median, the weighted mode, and the simple mode are shown. The slope of each straight line indicates the magnitude of the causal association. SNP, single nucleotide polymorphism; SE, standard error.

The IVW method analysis showed that the habit of consuming dried fruits (OR: 0.065, 95% CI: 0.008 ~ 0.555, *p* = 0.012) has a causal effect on psoriasis, indicating a negative correlation. Regular consumption of dried fruits can reduce the risk and severity of psoriasis. However, the *p*-values for the MR-Egger and WME method analyses were greater than 0.05, thus lacking statistical significance (Table 3). The *p*-values obtained from Cochran’s Q test for the IVW and MR-Egger methods were 0.0003 and 0.0002, respectively, both less than 0.05, indicating some heterogeneity in the analysis results, but with minor impact on the IVW results. The p-value for the intercept obtained using the MR-Egger method was 0.941, suggesting no horizontal pleiotropy. After sequentially excluding each SNP using the leave-one-out approach, the effect sizes of the remaining SNPs did not significantly differ from the overall effect size, indicating the absence of SNPs with a significant impact on the results, as shown in Figure 3. The forest plot, funnel plot, and scatter plot obtained from the MR analysis are shown in Figures 4–6, respectively.

**Table 3:** MR analysis for the causality of psoriasis with the risk of diet habits.

Exposure	Outcome	nSNP	Methods	b	se	*p*- val	OR (95%CI)	Horizontal pleiotropy			Heterogeneity Cochran’s	
								Egger intercept	se	*p*- val	Q	*p*
GCST90096916	Psoriasis	69	Inverse variance weighted	−2.730	1.092	0.012	0.065 (0.008–0.555)				115.125	0.0003
			MR Egger	−2.479	3.544	0.487	0.084 (0.000–87.150)	−0.001	0.009	0.941	115.115	0.0002
			Weighted median	−2.326	1.410	0.084	0.098 (0.007–1.370)					
Psoriasis	GCST90096916	17	Inverse variance weighted	0.001	0.004	0.895	1.001 (0.992–1.009)					
			MR Egger	0.027	0.017	0.145	1.027 (0.993–1.063)					
			Weighted median	0.002	0.005	0.681	1.002 (0.993–1.011)					

*p-val, p-value; OR, odds ratio; CI, confidence interval.*

## 4 Discussion

Epidemiological surveys indicate that from 1990 to 2019, the global incidence of psoriasis has shown an upward trend with increasing age, with a notable rise anticipated in the next decade among the 30–49 age group (13). Identifying potential risk and protective factors, such as dietary consumption habits, is crucial in preventing the onset and progression of psoriasis against a backdrop of genetic factors. This study utilized a two-sample MR approach to analyze the GWAS database, investigating the relationship between 29 dietary consumption habits and psoriasis. Previous literature has confirmed the correlation between psoriasis and dietary factors. However, to our knowledge, this is the first study to investigate the causal relationship between dietary consumption habits and psoriasis. The results show that among the 29 dietary habits analyzed, the intake of dried fruits has a causal relationship with psoriasis, with increased consumption acting as a protective factor against the disease.

In our study, we observed a causal relationship between increased intake of dried fruits and the alleviation of psoriasis symptoms. We believe this is primarily related to the following three factors:

Firstly, an increase in dried fruit consumption can effectively reduce calorie intake while ensuring sufficient energy. Previous studies have shown that low-calorie diets and intermittent fasting are effective interventions for alleviating psoriasis. These dietary approaches can decrease levels of interleukin-6 (IL-6), vascular cell adhesion molecule-1 (VCAM-1), intercellular adhesion molecule-1 (ICAM-1), and low-density lipoprotein (LDL) in patients with psoriasis, thereby suppressing skin inflammation and abnormal keratinization (14, 15). A secondary analysis of the National Health and Nutrition Examination Survey indicated that dried fruit consumers had significantly lower average body mass index (BMI) (−0.8, 99% CI -1.4 to −0.2; *p* = 0.002) and waist circumference (−2.6 cm, 99% CI -4.2 to −0.9 cm; *p* < 0.001) compared to non-consumers (16, 17). With the same caloric content, dried fruits provide more satiety compared to liquids and other fresh fruits and vegetables, leading to a decrease in appetite and a reduction in energy intake, thus preventing an increase in inflammatory mediators such as secreted phosphoprotein 1 (SPP1) and IL-17 due to weight gain (18). Additionally, dried fruits have long been a crucial component of the Mediterranean diet (19). A European observational cross-sectional study demonstrated a significant inverse association between the Mediterranean diet and the risk of psoriasis (OR: 0.34, 95% CI: 0.13–0.92, *p* = 0.03). Psoriasis patients who adopted a Mediterranean diet showed improvements in both BMI and PASI scores (20, 21).

Secondly, dried fruits are rich in dietary fiber (22), which can modulate the gut microbiota of patients, influencing their lipid and glucose metabolism and immune homeostasis, thereby reducing the severity of psoriasis and delaying its progression (23). Tian et al. found (24) that adding dried goji berries to the diet can increase the abundance of gastrointestinal lactobacilli and bifidobacteria, both of which are major components of probiotic therapy for psoriasis. The gut microbial community, through its metabolites entering the bloodstream and accumulating in the skin, regulates levels of skin inflammation factors, thus affecting the development of psoriasis (25).

Finally, dried fruits are rich in antioxidants and bioactive substances that can mitigate oxidative stress damage. The nutritional content of dried fruits is comparable to that of fresh fruits; however, the drying and concentration process results in an increase in polyphenol content, thereby enhancing the antioxidant activity of dried fruits more than that of fresh fruits (26–28). A prospective study in Romania showed that patients with psoriasis exhibited elevated levels of reactive oxygen species and free radical test levels. After undergoing psoriasis treatment, the oxidative stress levels in patients decreased but still did not return to normal ranges (29), although this conclusion still requires experimental research for support. Additionally, the high content of vitamins A, C, and E in dried fruits can reduce the severity of psoriasis by interacting with free radicals and preventing oxidative damage to macromolecules such as low-density lipoproteins (30).

The strength of this study lies in establishing a causal relationship between the dietary habit of dried fruit consumption and psoriasis. Consuming dried fruits does not replace the intake of other fruits; rather, it increases the total fruit intake (17) due to its satiating effect and minimal impact on metabolism (such as lipids and blood sugar), aligning with the patterns of a healthy diet. Therefore, for patients with psoriasis, increasing the intake of dried fruits could be an effective strategy to simultaneously boost the intake of bioactive substances, fiber, and trace elements, reducing the severity of psoriasis. However, evidence of the underlying mechanisms is limited, and further experimental and clinical studies are needed to validate these conclusions.

This study still has limitations. All research subjects were derived from European samples and do not represent a truly random population sample, thus the generalizability to other ethnic groups remains unclear, and there is a potential for overlap within the sample population. Although various sensitivity analyses were conducted to test the assumptions of the MR study, it is challenging to completely eliminate pleiotropy in the instrumental variables. Additionally, Steiger filtering and MR-PRESSO filtering are also helpful to mitigate horizontal pleiotropy. Moreover, the study’s findings are primarily based on conclusions drawn from the IVW method, with results from MR-Egger and the WME methods not being significant, which still raises concerns about the robustness of the study results. Additional GWAS dataset are expected to perform repeated validation in order to further confirm the causal relationship between dried fruit intake and psoriasis, such as Meta-analysis, or MVMR analyses, in order to examine whether dietary consumption habits impacts psoriasis by working through other factors. Additionally, this study did not find a causal relationship between the consumption of red wine, beer, vodka, and other dietary habits and psoriasis, despite previous research indicating these as risk dietary factors for psoriasis, a more comprehensive catalog database is needed for further research. The number of available items in the dietary questionnaire in the GWAS catalog database is limited, hence only existing effective and specific food groups could be studied as exposure factors, and the focus remains on the genetic influences on food groups or dietary patterns rather than more specific foods.

Overall, this study identifies the consumption of dried fruits as a protective factor against psoriasis, providing a basis for further research to refine and identify genetic markers associated with specific foods, thereby facilitating more precise analyses of the impact of dietary factors on psoriasis.

## 5 Conclusion

In summary, this study finds that increasing the intake of dried fruits may act as a protective factor against psoriasis. This result could serve as a reference for further exploration of healthy dietary patterns for patients with psoriasis.

## Data Availability

Publicly available datasets were analyzed in this study. This GWAS data for dietary consumption can be found: https://ftp.ebi.ac.uk/pub/databases/gwas/summary_statistics/GCST90096001-GCST90097000/GCST90096892/ and the data for psoriasis analysis can be found: https://r10.finngen.fi/pheno/L12_PSORIASIS.

## Footnotes

^1^ https://www.ebi.ac.uk/gwas/

^2^ https://www.finngen.fi/en
